# Subacute infective endocarditis due to *Lodderomyces elongisporus*: a case report and review of the literature

**DOI:** 10.3389/fpubh.2023.1181377

**Published:** 2023-10-20

**Authors:** Ye Qiu, Yongying Shi, Ying Mai, Zhile Wu, Jing Guan, Juanni Huang, Danhong Su, Feng Ye, Zhengtu Li

**Affiliations:** ^1^State Key Laboratory of Respiratory Disease, National Clinical Research Center for Respiratory Disease, Guangzhou Institute of Respiratory Health, The First Affiliated Hospital of Guangzhou Medical University, Guangzhou, China; ^2^Geriatric Department, National Clinical Research Center for Respiratory Disease, Guangzhou Institute of Respiratory Health, The First Affiliated Hospital of Guangzhou Medical University, Guangzhou, China; ^3^Microbiology Laboratory, The First Affiliated Hospital of Guangzhou Medical University, Guangzhou, China

**Keywords:** *Lodderomyces elongisporus*, infective endocarditis, central nervous system infection, invasive fungal disease, fungemia

## Abstract

*Lodderomyces elongisporus*, a rare emerging pathogen, can cause fungemia often related to immunosuppression or intravenous devices. Herein, we report the case of a 58-year-old woman with subacute infective endocarditis due to *Lodderomyces elongisporus* identified by blood fungal culture and whole-genome sequencing, who was treated with antifungals, mitral replacement and endocardial vegetation removal surgery.

## Case report

**Figure 1:**
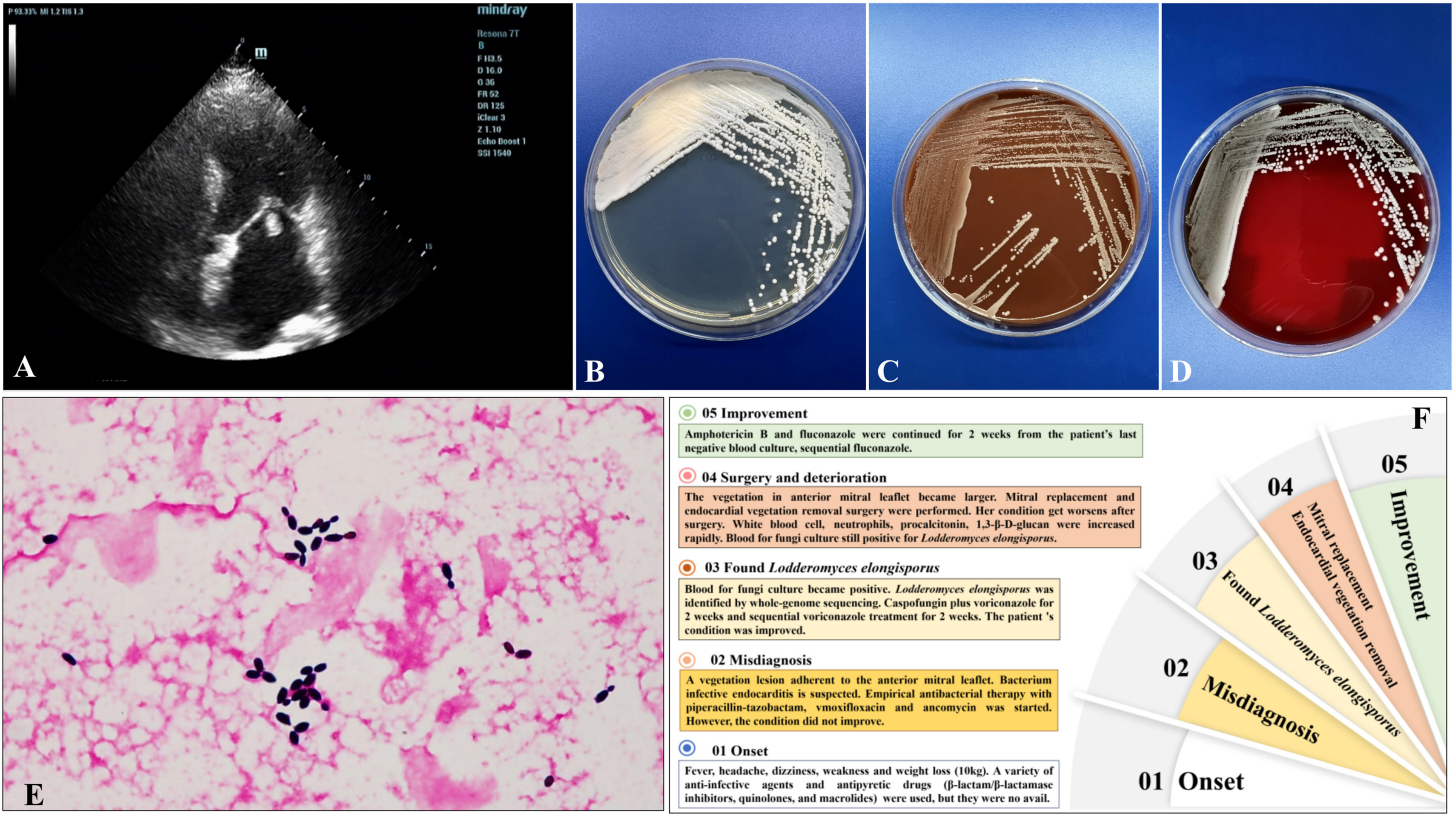
**(A)** Transthoracic echocardiography showed a vegetation lesion adherent to the patient's anterior mitral leaflet, which was concerning for infectiousendocarditis. **(B–D)** Culture and microscopic characteristics of *L. elongisporusfrom* the patient. Blood culture growth of cream-colored colonies on Sabouraud dextrose agar **(B)**, chocolate agar **(C)**, and Columbia CNA bloodagar plates **(D)** after 24 h of incubation at 35°C. **(E)** Microscopy of the blood smear revealed ellipsoidal to elongated blastoconidia of *L. elongisporus* (Gram stain, × 1,000). **(F)** The patient's disease development process.

A 58-year-old woman was admitted to our hospital on September 8, 2022 (Day 1) with a 2-month history of repeated low fever, headache, dizziness, weakness in both lower limbs and excessive weight loss (10 kg). Before admission, she had been given a variety of anti-infective agents and antipyretic drugs (β-lactam/β-lactamase inhibitors, quinolones, and macrolides) at the local hospital to no avail. The patient gave no recent travel history. She had a history of rheumatoid arthritis without treatment, type 2 diabetes, and hypertension. This patient did not have intravenous devices or any complications of diabetes. Body temperature was 38.8°C, a systolic blowing noise was audible in the mitral valve auscultation area, and no superficial lymph nodes in the body were found. Laboratory tests showed a normal white blood cell count of 6.86 × 10^9^/L (normal 3.5–9.5 × 10^9^/L), with 67.2% neutrophils, rheumatoid factor (13.2 IU/mL, normal) and anti-streptolysin O (127.0 IU/mL, normal). Random blood glucose (19.45 mmol/L, normal <7.8), high-sensitivity C-reactive protein (33.96 mg/L; normal 0–5 mg/L), erythrocyte sedimentation rate (78 mm/h; normal 0–20), procalcitonin (0.30 ng/L; normal 0–0.1), and 1,3-β-D-glucan (245.33 pg/mL; normal 0–100) were elevated. Low levels of CD3^+^CD45^+^ T lymphocytes (919 cells/μL; normal 955-2860), CD3^−^CD19^+^ B lymphocytes (103 cells/μL; normal 95-560), CD3^−^CD16^+^CD56^+^ NK cells (35 cells/μL; normal 150–1,100), and CD3^+^CD16^+^CD56^+^ NKT cells (20 cells/μL; normal 40-300) were observed. The Aspergillus antigen test for serum galactomannan, CrAg lateral flow assay, T-SPOT TB, and parasite and tumor marker tests were negative. No other primary immunodeficiency diseases, human immunodeficiency virus infection, or antibody spectrum of autoimmune diseases was detected. Transthoracic echocardiography showed a vegetation lesion adherent to the patient's anterior mitral leaflet, which was concerning for infectious endocarditis (Day 3) (Figure 1A). Chest computed tomography (CT) revealed left atrial enlargement. Bacterial infective endocarditis was suspected. The patient was started on empirical antibacterial therapy with piperacillin-tazobactam, moxifloxacin and vancomycin. However, the patient's condition did not improve. Five days into the patient's hospital stay, her blood was positive for fungal culture (Day 5) (Figures 1B–E). Using whole-genome sequencing, the organism was identified as *Lodderomyces elongisporus* (*L. elongisporus*) (Supplemental Figure 1) from the blood. Antifungal susceptibility testing showed that *L. elongisporus* is sensitive to fluconazole, flucytosine, itraconazole, caspofungin, amphotericin B and voriconazole (Supplementary Table 1). Then, anti-infectious therapy was increased to caspofungin plus voriconazole for 2 weeks and sequential voriconazole treatment for 2 weeks. Subsequently, the patient's symptoms, including fever, headache, dizziness, and weakness, improved. However, transthoracic echocardiography showed that the vegetation lesion in the anterior mitral leaflet became larger. Thus, mitral replacement and endocardial vegetation removal surgery were performed (Day 30).

The patient's condition worsened after surgery. The white blood cell count of 14.89 × 10^9^/L (normal 3.5–9.5) with 78.4% neutrophils, procalcitonin (3.57 ng/L; normal 0-0.1), and 1,3-β-D-glucan (393.46 pg/mL; normal 0–100) increased rapidly. Blood for fungal culture was still positive for *L. elongisporus* (Day 39), and the result of antifungal susceptibility testing was the same as before. Then, liposomal amphotericin B (5 mg/kg.d, intravenous drip) was administered for 2 weeks, and fluconazole (400 mg/d, per os) continued for 4 weeks. The patient's blood culture turned negative, and the symptoms and signs improved observably (Day 57). After discharge from the hospital (Day 73), the patient received fluconazole for sequential antifungal therapy for the duration of the study (Figure 1F).

## Systematic literature review search strategy

We searched PubMed, Web of Science, Embase, and BIOSIS Library for original case reports and cohort studies published in English on *Lodderomyces elongisporus* (*L. elongisporus*) between January 1, 1985, and December 31, 2022. We used the keywords “*Lodderomyces elongisporus*” and “*L. elongisporus*”. The references of the retrieved articles were reviewed for additional relevant citations. The abstracts of all identified articles were viewed, and the full-text versions of relevant articles were retrieved for data extraction and analysis.

The inclusion criteria for articles and cases in the systematic literature review were as follows: (1) original case reports or cohort studies on *L. elongisporus* published in English between January 1, 1985, and December 31, 2022; and (2) articles describing definitive etiological evidence for *L. elongisporus* based on pathological and culture proof.

## Clinical outcome definitions

The clinical course of infection was divided into the following two categories: (1) survival (complete or partial improvement of clinical symptoms after antifungal treatment); and (2) death.

## Data extraction

The following data were extracted: geographical distribution, demographics, pathogen proof, clinical signs and symptoms, involvement sites, diagnosis, treatment, outcomes and minimum inhibitory concentration (MIC) values for antifungal drugs for *L. elongisporus*. If a case was reported in more than one publication, the most recent article was used for data extraction.

## Metagenomic next generation sequencing (mNGS) and analysis

The high-quality reads were assembled using the SPAdes software, version 3.15.2 (1), default pipeline. The genome assembly was evaluated by QUAST software, version 5.0.2. The completeness of genome assemblies was evaluated in BUSCO software, version 5.4.3 (2), against the database of fungi_odb10 (or saccharomycetes_odb10) (OrthoDB; https://www.orthodb.org). Genome annotations were performed using Funannotate software, version 1.8.13 (3). The sequencing data have been uploaded to NCBI (PRJNA954074).

## Discussion

*L. elongisporus*, a rare emerging pathogen that is an ascomycetous yeast, can cause fungemia often related to immunosuppression or intravenous devices (4). To date, 23 invasive *L. elongisporus* infection patients have been reported, including 22 with fungemia and 1 with meningitis (Table 1) (4–16). Notably, only one patient was a neonate, and the other 22 cases occurred in adult patients. In addition to bloodstream infections, *L. elongisporus* was also present in the central nervous system and intracranial vascular system in 7 patients. Only 3 patients (patients 1 and 11 in the systematic literature review and the present patient) had infective endocarditis (4, 14). All 3 patients presented with cardiac valve vegetations adherent to the prosthetic aortic valve, anterior aorta and mitral valve. Two patients were diagnosed with blood-positive fungal cultures and identified as having *L. elongisporus* by PCR amplification or PCR amplification, followed by DNA sequencing (PCR sequencing) of the internal transcribed spacer (ITS) region and/or the D1/D2 domains of ribosomal ^®^DNA or by matrix-assisted laser desorption ionization time-of-flight (MALDI-TOF). In our present patient, diagnosis with blood-positive fungal culture and identification of *L. elongisporus* was achieved through mNGS.

**Table 1:** Demographic and clinical characteristics of 23 cases of *Lodderomyces elongisporus* fungemia.

**ID**	**Sex/age**	**Region**	**Underlying disease/risk factors**	**Infection signs/symptoms**	**CNS symptoms/signs**	**Isolation specimen**	**Method of identification**	**Diagnosis**	**Treatment**	**Prognosis**
P1 (4)	M/46y	USA	Injection drug Aortic valve replacement HCV	Chest pain Vegetations adherent to prosthetic aortic valve	Unresponsive Intracerebral hemorrhage with intraventricular bleeding	Blood	Culture MALDI-TOF PCR sequencing	Disseminated infection leading to endocarditis and artery mycotic aneurysm	Embolization L-AMB + 5-FC continued for 6 weeks	Survive
P2 (5)	F/9d	Kuwait	Premature neonates^*^ HMD Mechanical ventilation Invasive procedures	Fever	Choroid plexus cyst Intraventricular bleeding	Blood	Culture PCR sequencing	Fungemia	L-AMB	Death
P3 (6)	M/62y	Canada	Carcinoma of the rectum Hypertension Hyperlipidaemia Chronic lymphopenia^#^		Headache, dizziness, gait ataxia, urinary incontinence, confusion, hemiparesis, hydrocephalus	Arachnoid biopsy	Pathology PCR sequencing	Meningitis	AMB+VOR followed by FLU for 13 months Ventriculopleural shunt	Survive
P4 (7)	F/54y	Australia	Central venous catheter Total colectomy and ileostomy Stoma and TPN	Fever	NONE	Blood	Culture MALDI-TOF	Fungemia	AFG for 20 days	Survive
P5 (8)	F/71y	Kuwait	Hypertension Ischaemic heart disease Peripheral vascular disease Stroke	Septic shock	Unconscious	Blood	Culture PCR sequencing	Fungemia	CAS	Death
P6 (9)	F/56y	Korea	Lung cancer and lung lobectomy Central venous catheter High-dose dexamethasone Cord decompression surgery Broad-spectrum antibiotics	Dyspnoea and fever WBC and CRP increased Pleural effusion with multiple lung nodules	NONE	Blood	Culture MALDI-TOF PCR sequencing	Catheter-related fungemia	Not mentioned	Death
P7 (10)	M/39y	Japan	Aorto-oesophageal fistula Thoracoabdominal aortic replacement	Fever	NONE	Blood Catheter tip	Culture PCR sequencing	Catheter-related fungemia	Blood culture turn negative after use MFG 72 h MFG 100 mg/d for 42 days	Survive
P8 (11)	M/79y	Spain	COPD Diabetes melitus ESRD	Sepsis	NONE	Blood	Culture PCR sequencing	Fungemia	CAS 3 days	Death
P9 (12)	M/22y	Qatar	Trauma victim	Not mentioned	Not mentioned	Blood	Culture PCR sequencing MALDI-TOF MS	Fungemia	CAS, FLU	Death
P10 (13)	M/63	Kuwait	Schizophrenic-like condition Nasogastric tube Central venous catheter	Fever Hypotension Bradycardia	Unconscious, confused, disoriented, slurred speech, diminished reflexes, lacunar infarcts, and left hemiparesis with seizure.	Catheter Tip Blood	Culture PCR sequencing	Fungemia	FLU 400 mg/day for 10 days Blood culture turn negative	Survive
P11 (14)	M/30	Australia	Depression and claustrophobia Injection drug	Fever, night sweats and rigors Recurrent swelling of left forefoot Cardiac murmur Vegetations attached to anterior aortic	Right frontal and left parietal embolic lesions	Blood Aortic valve vegetations	Culture, Pathology PCR sequencing	Endocarditis Osteomyelitis	Blood culture turns negative after CAS for 48 h CAS for 2 weeks, followed by AMB+5-FU for 6 weeks, then sequential VOR for 7.5 months Mechanical aortic valve.	Survive
P12 (15)	–	Malaysia	Not mentioned	Not mention	Not mentioned	Blood	Culture PCR sequencing	Fungemia	Not mentioned	Not mentioned
P13-P22 (16)	4 M/6 F 1–79y (median 55y)	1 Malaysia 1 China 8 Mexico	Not mentioned	Not mention	Not mentioned	Blood	Culture, PCR sequencing	Fungemia	Not mentioned	1 Death 9 Survive
P23^**^	F/58y	China	Rheumatoid arthritis Diabetes melitus Hypertension Broad-spectrum antibiotics	Fever, weakness Vegetations adherent to mitral valve	Headache, dizziness	Blood	Culture, mNGS	Endocarditis Fungemia	ASP+VOR for 2 weeks, sequential VOR for 2 weeks. AMB + FLU for 2 weeks	Survive

^**^P23 was the present case.

^*^An extremely low birth weight (ELBW, 710 g), preterm (23 weeks of gestational age), cannulated with an umbilical arterial catheter and an umbilical venous catheter using a vacuum-assisted closure technique and started on total parenteral nutrition.

^**#**^Chronic lymphopenia: lymphocyte count <0.3 × 109 g/L. HMD, hyaline membrane disease; HCV, hepatitis C virus; LE, *Lodderomyces elongisporus*; TPN, total parenteral nutrition; CNS, Central nervous system; ESRD, End-stage renal disease; COPD, chronic obstructive pulmonary disease. PCR amplification or PCR amplification followed by DNA sequencing (PCR sequencing) of the internal transcribed spacer (ITS) region and/or the D1/D2 domains of ribosomal ^®^DNA or by matrix-assisted laser desorption ionization time-of-flight mass spectrometry (MALDI TOF-MS).

FLU, fluconazole; VOR, voriconazole; ITR, itraconazole; POS, posaconazole; AFG, anidulafungin; MFG, micafungin; CAS, caspofungin; AMB, amphotericin B; 5-FC, 5-fluorocytosine; ASP, aspofungin.

In this case report and review of the literature, only 2 patients were not clearly identified for the existence of underlying disease/risk factors (15, 16). The underlying disease/risk factors were as follows: surgery and trauma (6 patients), cardiovascular and cerebrovascular diseases (6 patients), central venous catheterization (3 patients), injection drugs (2 patients), cancer (2 patients), diabetes mellitus (2 patients), broad-spectrum antibiotics (2 patients), and mental illness (2 patients). Each case of these diseases included high-dose dexamethasone, nasogastric tube, stoma and total parenteral nutrition, mechanical ventilation, aorto-esophageal fistula, end-stage renal disease, rheumatoid arthritis, premature neonates, hyaline membrane disease, chronic lymphopenia, hepatitis C virus and chronic obstructive pulmonary disease. These results suggested that, in addition to the previously discussed immunosuppression and intravenous devices underlying diseases, surgery and trauma, cardiovascular and cerebrovascular diseases, cancer, diabetes mellitus and broad-spectrum antibiotics, vigilance against *L. elongisporus* infection is also needed.

There is currently a lack of consensus and guidelines for recognized treatment of *L. elongisporus* infection. The crude mortality rate in this study for invasive *L. elongisporus* infection was 27.27% (6/22). The minimum inhibitory concentration values for 23 strains of *L. elongisporus* indicated that they were sensitive to common antifungal drugs. It is worth noting that, when regular antifungal therapy for *L. elongisporus* endocarditis is not effective, surgical intervention should be considered. In addition, it is essential to incorporate prospective future projects or study subjects pertaining to the proper identification of *L. elongisporus*, as well as risk factors, surveillance, epidemiological investigations and standardized diagnosis and treatment.

In this study, we utilized the mNGS method for pathogen detection in a particular sample. The high-quality reads obtained were assembled using the default pipeline of SPAdes software, version 3.15.2 (1). We evaluated the genome assembly using QUAST software, version 5.0.2, and assessed the completeness of the genome assembly using BUSCO software, version 5.4.3 (2), against the fungi_odb10 (or saccharomycetes_odb10) database from OrthoDB (OrthoDB | E Zdobnov lab). We performed genome annotations using Funannotate software, version 1.8.13 (3).

To determine the closest match to the sample, we aligned the assembled scaffolds to the abvf (archaea, bacteria, viruses, fungi) database from the NT and ITS databases. In both cases, the results showed that the sample was closest to *Lodderomyces elongisporus*. To confirm our conclusion, we calculated the values of ANI (average nucleotide identity) and dDDH (digital DNA–DNA hybridization) among the species, which also indicated a close match to *Lodderomyces elongisporus*.

Furthermore, we used reads to map the abvf database directly with blastn (17) and Kraken (18)-Bracken (19). The classification results revealed that most of the reads were labeled *Lodderomyces elongisporus*, providing additional support for our conclusion.

We conducted a phylogenetic analysis to determine the relationships of the sample *L. elongisporus* in the Saccharomycetales order. The results provided important insights into the evolutionary relationships of *L. elongisporus* within the Saccharomycetales order and its position among other fungi. This information could be useful for understanding the genetic diversity and evolutionary history of fungal species, as well as for developing effective classification schemes and identifying potential targets for drug development.

The use of mNGS technology in clinical pathogen detection demonstrated high accuracy in detecting fungi. This technology allowed for a comprehensive and precise analysis of the sample's genome assembly, as well as identification of the closest match to *Lodderomyces elongisporus*. The findings suggested that mNGS has great potential as a valuable tool for clinical pathogen detection and research.

The results of our study suggested that mNGS could be a valuable tool for clinical pathogen detection and open new avenues for research in the field. Moreover, mNGS could provide important data on pathogen epidemiology and evolution for infectious disease surveillance. By analyzing large amounts of pathogen genomic data, a deeper understanding of the pathogen's evolutionary history, spread pattern, and interaction with the host can be achieved. This information is significant for developing effective prevention and control strategies and researching new vaccines.

## Data availability statement

The original contributions presented in the study are included in the article/Supplementary material, further inquiries can be directed to the corresponding authors.

## Ethics statement

The Ethics Review Board of the First Affiliated Hospital of Guangzhou Medical University (reference number 2022051) approved the study, which was conducted in accordance with Good Clinical Practice and the Declaration of Helsinki. Written informed consent was obtained from the individual(s) and/or minor(s)' legal guardian/next of kin for the publication of any potentially identifiable images or data included in this article.

## Author contributions

YQ, ZL, and FY were in charge of writing and editing this manuscript. YM, JG, and DS were responsible for the etiological examination and antimicrobial susceptibility test. YS, ZW, and JH were responsible for the collection and follow-up of the case data. All authors contributed to the article and approved the submitted version.

## Supplementary material

Supplementary Figure 1 Phylogenetic tree of Lodderomyces elongisporus (sample) in the Saccharomycetales order, using representative genome strains from the RefSeq database (ftp://ftp.ncbi.nlm.nih.gov/refseq/) and an outgroup species Trichoderma of ascomycetes. The tree was constructed based on single-copy genes, and the series of values over the branches corresponds to ML bootstrap values.

Supplementary Figure 2 Antimicrobial susceptibility testing of minimum inhibitory concentration values (μg/ml) for the L. elongisporus strain by Etest in a fungal susceptibility plate (Jiangmen Kailin Trading Co., Ltd.) in 35 °C for 48 hours.
