# Supplementary figures and images for: Subacute infective endocarditis due to Lodderomyces elongisporus: a case report and review of the literature
Source: Front Public Health. 2023 Oct 20;11:1181377. doi: 10.3389/fpubh.2023.1181377 (PMC10624219; doi:10.3389/fpubh.2023.1181377)

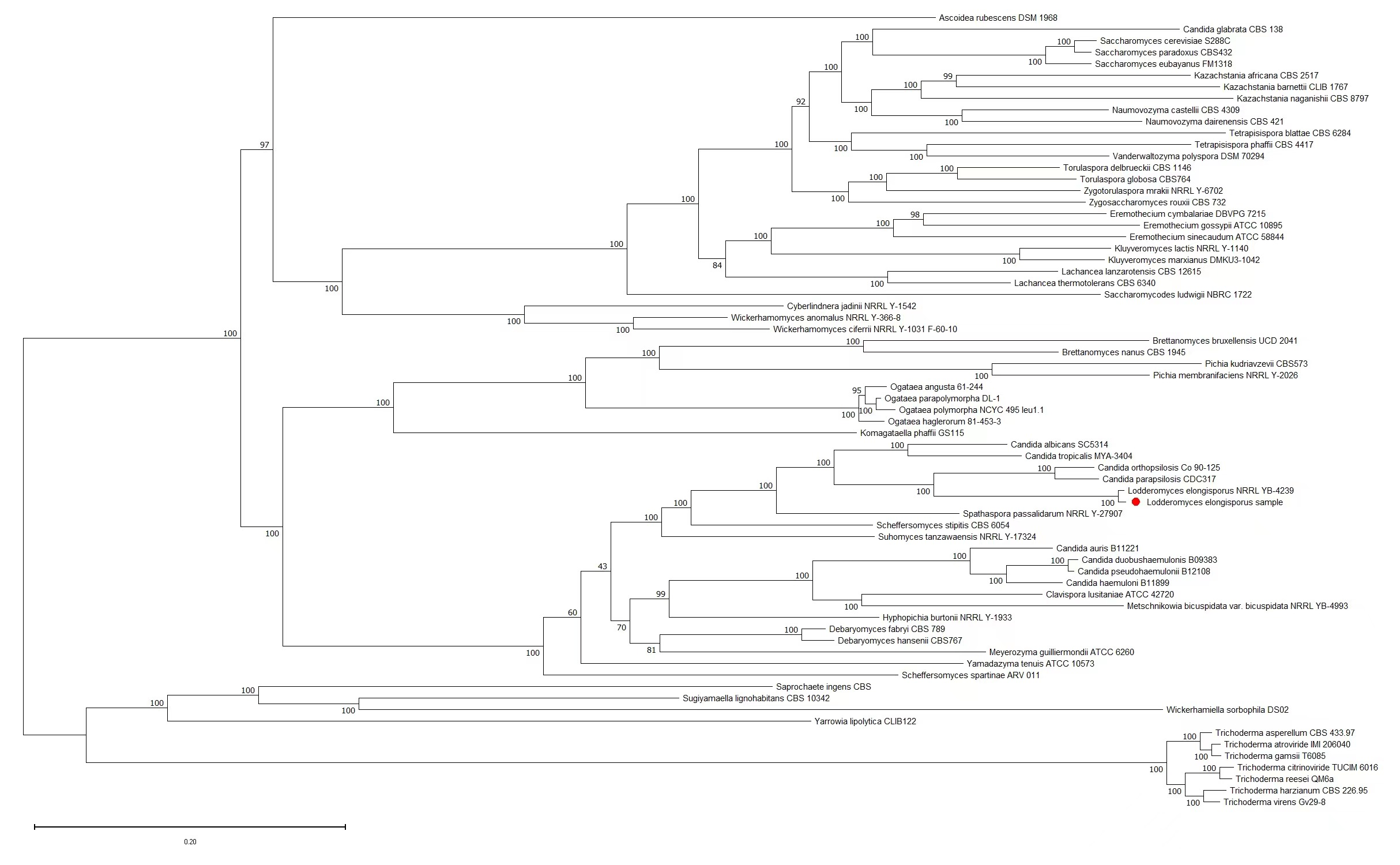

Supplement: Supplementary Figure 1 — Phylogenetic tree of Lodderomyces elongisporus (sample) in the Saccharomycetales order, using representative genome strains from the RefSeq database (ftp://ftp.ncbi.nlm.nih.gov/refseq/) and an outgroup species Trichoderma of ascomycetes. The tree was constructed based on single-copy genes, and the series of values over the branches corresponds to ML bootstrap values. [file Image_1.tif]

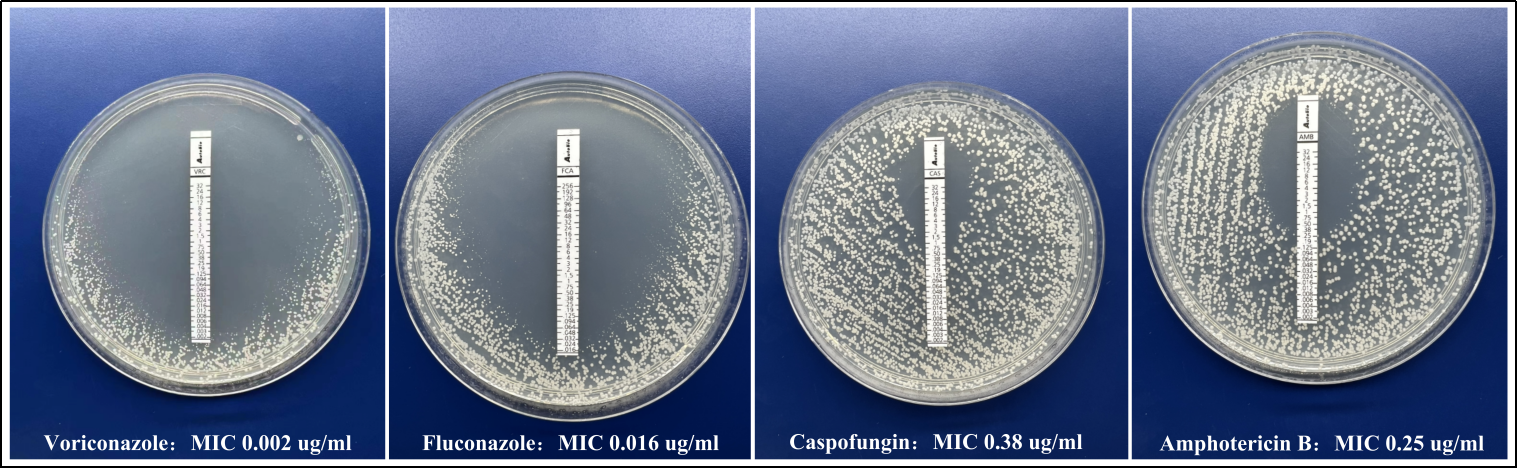

Supplement: Supplementary Figure 2 — Antimicrobial susceptibility testing of minimum inhibitory concentration values (μg/ml) for the L. elongisporus strain by Etest in a fungal susceptibility plate (Jiangmen Kailin Trading Co., Ltd.) in 35 °C for 48 hours. [file Image_2.tif]
