# Supplementary material for: Subacute infective endocarditis due to Lodderomyces elongisporus: a case report and review of the literature
Source: Front Public Health. 2023 Oct 20;11:1181377. doi: 10.3389/fpubh.2023.1181377 (PMC10624219; doi:10.3389/fpubh.2023.1181377)
Supplement: Supplementary file 3 [file Table_1.docx]

**Online** **supplement for results**

**Subacute infective endocarditis due to *Lodderomyces elongisporus*: a case report and review of the literature**

**Supplemental Table 1. Minimum inhibitory concentration values (****µg/ml) for 23 strains of *L.* *elongisporus***

| **ID** | Fluconazole | Voriconazole | Itraconazole | Posaconazole | Anidulafungin | Micafungin | Caspofungin | Amphotericin B | 5-fluorocytosine | Isavuconazole |
| --- | --- | --- | --- | --- | --- | --- | --- | --- | --- | --- |
| **P1^[1]^** | 0.25 | 0.06 | 0.06 | 0.06 | ≤0.015 | 0.03 | 0.015 | ≤0.12 | 0.12 | ND |
| **P2^[2]^** | 0.25 | ≤0.008 | ≤0.03 | ≤0.008 | 0.02 | 0.02 | 0.03 | 0.13 | ≤0.06 | ND |
| **P3^[3]^** | ND | ND | ND | ND | ND | ND | ND | ND | ND | ND |
| **P4^[4]^** | 0.12 | 0.008 | 0.03 | 0.015 | 0.015 | 0.015 | 0.008 | 0.12 | 0.06 | ND |
| **P5^[5]^** | 0.125 | 0.004 | 0.008 | 0.003 | ND | 0.003 | 0.064 | 0.012 | 0.064 | ND |
| **P6^[6]^** | 1.00 | 0.12 | ND | ND | ND | 0.06 | 0.25 | 0.25 | 1.00 | ND |
| **P7^[7]^** | 0.5 | 0.25 | 0.015 | ND | ND | 0.015 | ND | 0.25 | 0.5 | ND |
| **P8^[8]^** | 0.125 | 0.0017 | ND | 0.007 | 0.015 | 0.015 | ND | 0.031 | ND | ND |
| **P9^[9]^** | 0.25 | <0.016 | 0.031 | 0.063 | 0.016 | ND | 0.5 | 0.5 | ND | <0.016 |
| **P10^[10]^** | 0.32 | 0.002 | ND | 0.023 | ND | ND | 0.094 | ND | 0.094 | ND |
| **P11^[11]^** | ≤0.125 | ≤0.008 | 0.06 | 0.03 | ND | ND | 0.03 | 0.25 | 0.06 | ND |
| **P12^[12]^** | 0.125 | 0.047 | 0.004 | ND | ND | ND | 0.015 | 0.012 | ND | ND |
| **P13^[13]^** | 0.12 | ND | ND | ND | 0.03 | 0.015 | 0.03 | 0.75 | ND | ND |
| **P14^[13]^** | 0.25 | ND | ND | ND | 0.03 | 0.015 | 0.03 | 0.75 | ND | ND |
| **P15^[13]^** | 0.25 | ND | ND | ND | 0.12 | 0.015 | 0.03 | 0.75 | ND | ND |
| **P16^[13]^** | 0.25 | ND | ND | ND | 0.12 | 0.03 | 0.03 | 0.50 | ND | ND |
| **P17^[13]^** | 0.25 | ND | ND | ND | 0.12 | 0.015 | 0.03 | 0.375 | ND | ND |
| **P18^[13]^** | 0.25 | ND | ND | ND | 0.015 | 0.015 | 0.03 | 0.375 | ND | ND |
| **P19^[13]^** | 0.25 | ND | ND | ND | 0.015 | 0.015 | 0.03 | 0.75 | ND | ND |
| **P20^[13]^** | 0.25 | ND | ND | ND | 0.015 | 0.015 | 0.03 | 0.5 | ND | ND |
| **P21^[13]^** | 0.25 | ND | ND | ND | 0.015 | 0.015 | 0.03 | 0.375 | NA | ND |
| **P22^[13]^** | ND | ND | ND | ND | ND | ND | ND | ND | ND | ND |
| **P23*** | 0.016 | 0.002 | ND | ND | ND | ND | 0.38 | 0.25 | ND | ND |

*P23 was the present case. ND: not done

**References**

1. Thompson CM, Warner N, Hurt CB, Alby K, Miller MB. Closing the Brief Case: A Case of Prosthetic Valve Endocarditis Due to *Lodderomyces elongisporus*. J Clin Microbiol. 2021 21;59(2):e01227-20.
2. Asadzadeh M, Al-Sweih N, Ahmad S, Khan S, Alfouzan W, Joseph L. Fatal *Lodderomyces elongisporus* Fungemia in a Premature, Extremely Low-Birth-Weight Neonate. J Fungi (Basel). 2022;8(9):906.
3. Dear T, Joe Yu Y, Pandey S, Fuller J, Devlin MK. The first described case of *Lodderomyces elongisporus* meningitis. J Assoc Med Microbiol Infect Dis Can. 2021;6(3):221-228.
4. Koh B, Halliday C, Chan R. Concurrent bloodstream infection with *Lodderomyces elongisporus* and Candida parapsilosis. Med Mycol Case Rep. 2020;28:23-25.
5. Al-Obaid K, Ahmad S, Joseph L, Khan Z. *Lodderomyces elongisporus*: a bloodstream pathogen of greater clinical significance. New Microbes New Infect. 2018;26:20-24.
6. Lee HY, Kim SJ, Kim D, et al. Catheter-related Bloodstream Infection due to *Lodderomyces elongisporus* in a Patient with Lung Cancer. Ann Lab Med. 2018;38(2):182-184.
7. Hatanaka S, Nakamura I, Fukushima S, Ohkusu K, Matsumoto T. Catheter-Related Bloodstream Infection Due to *Lodderomyces elongisporus*. Jpn J Infect Dis. 2016;69(6):520-522.
8. Fernández-Ruiz M, Guinea J, Puig-Asensio M, et al. CANDIPOP Project; GEIH-GEMICOMED (SEIMC) and REIPI. Fungemia due to rare opportunistic yeasts: data from a population-based surveillance in Spain. Med Mycol. 2017;55(2):125-136.
9. Taj-Aldeen SJ, AbdulWahab A, Kolecka A, Deshmukh A, Meis JF, Boekhout T. Uncommon opportunistic yeast bloodstream infections from Qatar. Med Mycol. 2014;52(5):552-6.
10. Ahmad S, Khan ZU, Johny M, et al. Isolation of *Lodderomyces elongisporus* from the Catheter Tip of a Fungemia Patient in the Middle East. Case Rep Med. 2013;2013:560406.
11. Daveson KL, Woods ML. *Lodderomyces elongisporus* endocarditis in an intravenous drug user: a new entity in fungal endocarditis. J Med Microbiol. 2012;61(Pt 9):1338-1340.
12. Minea B, Nastasa V, Moraru RF, et al. Species distribution and susceptibility profile to fluconazole, voriconazole and MXP-4509 of 551 clinical yeast isolates from a Romanian multi-centre study. Eur J Clin Microbiol Infect Dis. 2015;34(2):367-83.
13. Lockhart SR, Messer SA, Pfaller MA, Diekema DJ. *Lodderomyces elongisporus* masquerading as Candida parapsilosis as a cause of bloodstream infections. J Clin Microbiol. 2008;46(1):374-6.
